# The Impact of Air Pollution Controls on Health and Health Inequity Among Middle-Aged and Older Chinese: Evidence From Panel Data

**DOI:** 10.3389/ijph.2024.1606956

**Published:** 2024-06-14

**Authors:** Yaxin Zhao, Zixuan Peng, Zhongliang Zhou, Xiaohui Zhai, Shaoqing Gong, Chi Shen, Tianci Zhang, Dantong Zhao, Dan Cao

**Affiliations:** ^1^ School of Public Health, Health Science Center, Xi’an Jiaotong University, Xi’an, China; ^2^ School of Public Health, Southeast University, Nanjing, China; ^3^ School of Public Policy and Administration, Xi’an Jiaotong University, Xi’an, Shaanxi, China; ^4^ School of Public Health, Xi’an Jiaotong University, Xi’an, Shaanxi, China; ^5^ Luohe Medical College, Luohe, China; ^6^ College of Computing and Information Science, Cornell University, Ithaca, NY, United States

**Keywords:** air pollution controls, health, health inequality, China, difference-in-differences

## Abstract

**Objectives:**

We evaluated the long-term effects of air pollution controls on health and health inequity among Chinese >45 years of age.

**Methods:**

Data were derived from the China Health Aging and Retirement Longitudinal Survey and the China National Environmental Monitoring Centre. Decreases in PM_2.5_ and PM_10_ were scaled to measure air quality controls. We used a quasi-experimental design to estimate the impact of air quality controls on self-reported health and health inequity. Health disparities were estimated using the concentration index and the horizontal index.

**Results:**

Air pollution controls significantly improved self-reported health by 20% (OR 1.20, 95% *CI*, 1.02–1.42). The poorest group had a 40% (OR 1.41, 95% *CI*, 0.96–2.08) higher probability of having excellent self-reported health after air pollution controls. A pro-rich health inequity was observed, and the horizontal index decreased after air pollution controls.

**Conclusion:**

Air pollution controls have a long-term positive effect on health and health equity. The poorest population are the main beneficiaries of air pollution controls, which suggests policymakers should make efforts to reduce health inequity in air pollution controls.

## Introduction

Reducing the adverse effects of pollution is a critical component of several United Nations Sustainable Development Goals (SDGs), including but not limited to the goal of ensuring healthy lives and promoting wellbeing for all at all ages (Goal 3), reducing inequalities within and between countries (Goal 10) and promoting climate action (Goal 13) [1]. The World Health Organization (WHO) released the new Global Air Quality Guidelines (AQG) in 2021 to promote incremental improvements in air quality. The health effects and disease burden of air pollution are serious, and air pollution has been identified as one of the most urgent issues facing China [2–8].

A series of pollution control regulations and policies have been put in place to improve air quality [4, 8, 9], emphasizing environmental controls as a central component of the overall control plan in 2013, etc. These regulations and policies are both informal or formal [4], and command-and-control or market-based [10, 11]. In 2013, air pollution controls underwent significant changes on multiple fronts [12], and the government issued the most stringent Air Pollution Prevention and Control Action Plan in history to improve air quality by strengthening comprehensive controls [2, 7, 8, 13]. Evaluating the potential impact of air pollution control policies on health is of increasing interest to policymakers as they track progress toward achieving these SDGs and their respective impacts on health and health inequities.

There is substantial evidence that air pollution controls also have health benefits. Markandya et al. concluded that substantial health gains can be achieved by taking action to prevent climate change [14]. Yang and Chou found that shutting down a power plant in New Jersey reduced the likelihood of having a low-birth-weight baby by 15% [15]. Xie et al. proposed a cooperative reduction model that encourages neighboring areas to jointly control air pollution, saving 437 more lives than the non-cooperative reduction model [16]. Chamberlain concluded that low-emission zones have positive effects on air pollution-related health outcomes, especially cardiovascular disease [17]. Tonne found that the Congestion Charging Scheme can reduce levels of traffic pollutants, and has benefits in terms of increasing life expectancy and reducing socio-economic inequalities [18]. Although efforts have been made to take inequality into account when considering air pollution control interventions, studies about the impacts of air pollution controls on health inequality and health inequities among middle-aged and older populations in China are limited [19].

As the world’s most populous country, China plays a crucial role in current scientific and policy debates on the impact of air pollution controls on health inequities [20]. There is an urgent need to assess the long-term impacts of air pollution controls on health and health inequities in the context of improving air quality in China. Our study contributes to the extant research in three ways: first, we adopted a new way to measure air pollution controls In terms of whether or not they achieve the interval value between three targets assigned by the WHO, providing a new measurement for other developing countries that are also trying to achieve the targets listed by the WHO; second, we explored the long-term impact of air pollution controls on health outcomes by using the difference-in-differences approach to solving endogenous problems in policy evaluation; and finally, we estimated health inequality before and after air pollution controls by concentrate index and horizontal index to provide quantitative support for other low- and middle-income countries to reduce inequity in air pollution controls.

## Methods

### Study Design

Air pollution is caused by the presence of many different small substances in the air. In this study, we focus on two main pollutants: inhalable particulate matter with a diameter of 10 µm (PM_10_) (panel A) and fine particulate matter≤2.5 (PM_2.5_) (panel B). PM_10_ and PM_2.5_ are the primary air pollutants in the vast majority of cities in China, which are also the major air pollutants in the Global AQG released by the WHO. The health risks associated with particulate matter (PM₁₀ and PM_2.5_) are particularly important to public health. Both PM_2.5_ and PM₁₀ are capable of penetrating deep into the lungs. PM_10_ is often derived from resuspension and combustion products and causes damage to the human respiratory system and immune systems. PM_2.5_ is the primary air pollutant and has the most severe effects on human health.

The Chinese government has turned its attention to PM_2.5_ and PM_10_ and introduced a series of strict environmental policies to regulate air pollution. The standard concentration indicators for PM_2.5_ and ozone that China added to the Environmental Air Quality Standards (GB3095-2012) released in 2012 were based on the WHO 2005 AQG. Among them, PM_2.5_ is equivalent to the WHO-recommended first-stage transition target, which is an active attempt for China to align with international standards in air governance and plays a key role in China’s air pollution controls, promoting a significant improvement in air quality levels. Due to the increasingly prominent regional atmospheric environmental issues caused by PM_10_ and PM_2.5_ pollutants in China, the Chinese government issued the “Action Plan for Air Pollution Prevention and Control” in 2013, with the main goal of reducing PM_10_ and PM_2.5_ levels. According to the plan, PM_10_ levels in prefecture-level and above cities nationwide should have been reduced by more than 10% compared to 2012; PM_2.5_ in regions such as Beijing Tianjin Hebei, the Yangtze River Delta, and the Pearl River Delta should have been reduced by approximately 25%, 20%, and 15%, respectively, by 2017. Air pollution in China was severe before 2013, and some studies found that PM_2.5_ concentrations decreased in China’s major areas after 2013 [21]. Moreover, the Chinese government implemented the “Winning the Blue Sky Defense War Three-Year Action Plan” from 2018 to 2020, which required a further significant reduction of PM_2.5_. The air pollution regulations released in 2013 set the conditions for the design of the difference-in-differences technique.

**FIGURE 1:**
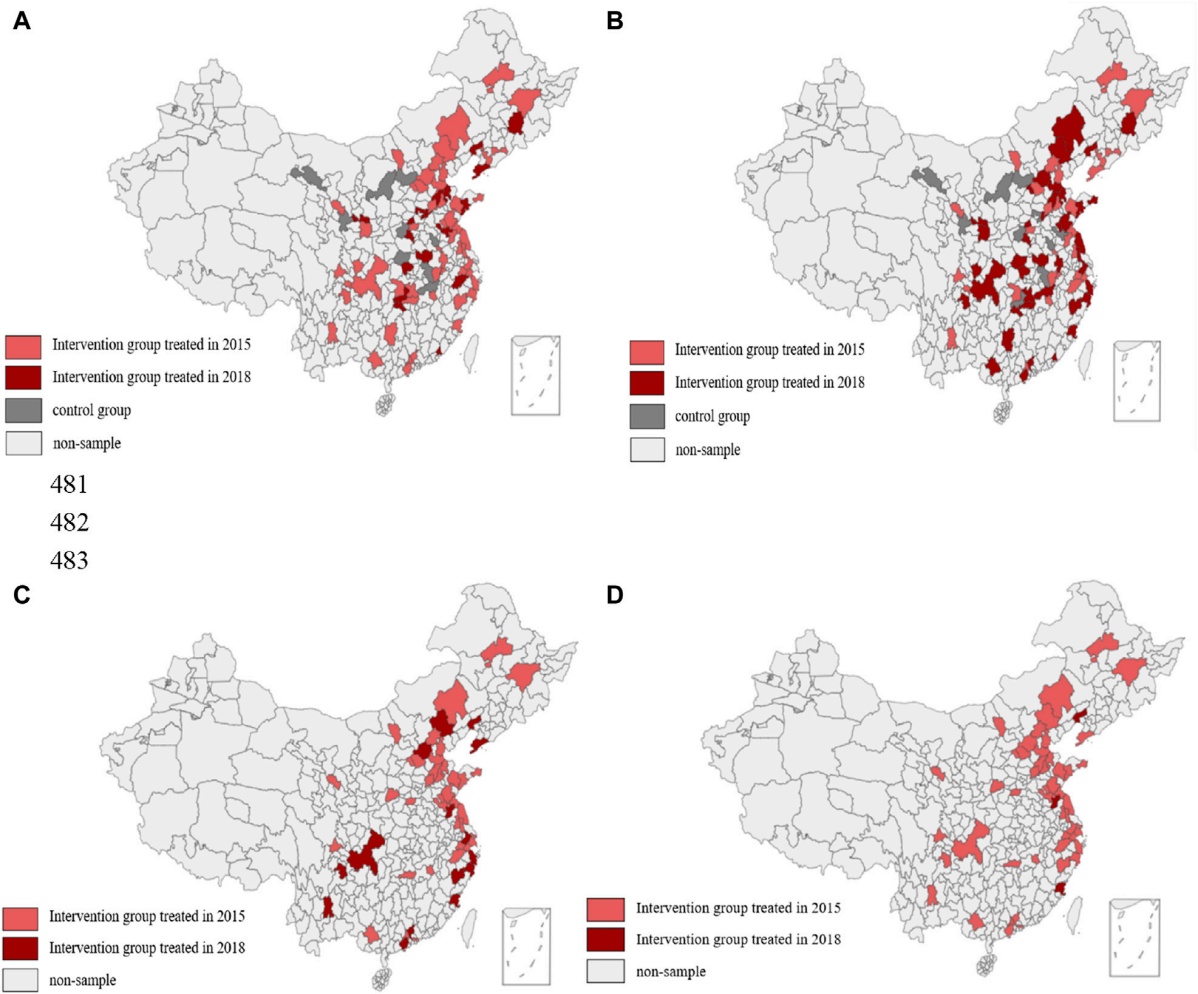
Geographical distribution of the cities in which the subjects were located for different air pollution control groups and different control times identified by PM_10_ and PM_2.5_ (Impact of Air Pollution Controls, Shaanxi, China, 2024). Note: **(A)** Shows the spatial distribution of cities for different groups and different times identified by PM_10_. **(B)** Shows the spatial distribution of cities for different groups and different times with a one-year lag identified by PM_10_. **(C)** Shows the spatial distribution of cities for different times identified by PM_2.5_. **(D)** Shows the spatial distribution of cities for different times with a one-year lag identified by PM_2.5_.

For developing countries, WHO developed three transitional criteria for PM_10_ and PM_2.5_ in 2005 and added the four-stage targets in 2021 (see Supplementary Tables S1, S2). The interval between the three stages for annual PM_10_ and PM_2.5_ was 20 ug/m^3^ and 10 ug/m^3^, respectively. Therefore, we used the decrease of 20 ug/m^3^ to measure the control of air pollution identified by PM_10_ (panel A), and the decrease of 10 ug/m^3^ to measure the control of air pollution identified by PM_2.5_ (panel B). If the city’s annual average concentration of PM_10_ and PM_2.5_ decreased by 20 ug/m^3^ and 10 ug/m^3^ above during the entire study period, it means that subjects living at this city treated by air pollution controls (treatment group). If the city’s annual average concentration of PM_10_ and PM_2.5_ increased or decreased slightly during the whole study period, it means that subjects living at this city have not been treated by air pollution controls (control group). There are different treatment groups and times for air pollution controls between panel A and panel B. The geographical distribution of the cities where the subjects were located for different air pollution control groups and different air pollution control times identified by PM_10_ and PM_2.5_ is shown in Figure 1.

### Data Sources

The individual-level health data come from the 2013, 2015, and 2018 China Health and Retirement Longitudinal Study (CHARLS), published by the National School of Development of Peking University. The CHARLS is a nationally representative longitudinal survey of individuals in China and their spouses, aged 45 years or older, covering 28 provinces, cities, and municipalities in the country and collecting assessments of the social, income, and health circumstances of community residents [22]. Air pollution data for each city between 2013 and 2018 were provided by the China National Environmental Monitoring Centre, the China Statistical Yearbook on the Environment, and a bulletin on the ecological environment of each province or city. The selection process is as follows: first, we delete the cities that missng the data of PM_10_ and PM_2.5_; and second, we delete subjects living in the cities that have met the WHO target with a low concentration during 2013–2018 and the cities that decreased in 2015 but increased in 2018 according to the difference-in-differences design; third, we delete the missing value for economic status and self-reported health; and finally, we drop the subjects that were not followed up in 2015 and 2018. After selection, we eventually obtained panel data A, including 19,446 participants in 82 cities who were followed up over the three waves of the surveys, and panel data B, including 12,171 participants in 51 cities.

### Variables

Dependent variables: self-reported health status. The answers are very good, good, fair, poor, and very poor. This reflects the overall subjective experience of mental and physical wellbeing and is closer to the WHO’s definition of health [5]. Moreover, self-reported health can be used as a valid predictor of mortality and other functional limitations in many countries and regions [14, 23, 24], We set the dependent variable as a binary variable. In this study, the description of the variable was taken as having a value of 1 if a subject chose very good or good. On the contrary, fair, poor, and very poor are classified as having a value of 0.

Independent variables: the dummy variable (Treat) indicates whether the city where the subjects lived is on the air quality control list. If the city where the subjects lived was on the air pollution control list between 2013 and 2018, its value was set as 1; otherwise, its value was set as 0. The dummy variable (Post) was set to 1 for the year of air pollution controls and the year after air pollution controls, and 0 for the year before air pollution controls. The core independent variable we were concerned with was the interaction term (Did and Did_t-1_) between the air pollution controls and the year dummy variables. Did is the net effect of air pollution control on health, and Did_t-1_ is the net effect of air pollution control with a one-year lag on health. Because we considered the effects of health lags and the time required to become aware of air quality, we used the annual average concentration of PM_2.5_ in the previous year as a proxy for the concentration in the current year.

Controls: the logarithm of gross domestic product (GDP) *per capita* for each city to represent the city-level indicator; we also controlled for individual-level indicators such as sex, age, work status, economic status, educational status, and health insurance.

### Statistical Analysis

The Difference-in-differences (DID) model with multiple periods and the two-way fixed-effects logistic regression model were used to estimate the causal effects of air pollution controls on self-reported health [25]. The use of quasi-experimental designs to evaluate the effects of policy treatments has gained wide acceptance in empirical research in the social sciences [26]. The DID method, which is widely accepted as the most common and best method for studying quasi-natural experiments [27] can control for temporal variation in the outcome that is not due to treatment exposure and the selection effect and has significant advantages in solving endogenous problems caused by causal identification and variable omission [28]. We compared average changes in health before and after the air pollution controls intervention between treatment and control cities [1]. It is well known that the DID analysis relies on the “common trend assumption,” which means that the DID estimator requires that the average outcomes for the treatment and control groups follow parallel paths in the pre-intervention periods. Moreover, robust analyses can be performed to evaluate whether the effect measured can be attributed to the introduction of air pollution controls. All analyses were conducted using the Stata software, version 14. The regression model is shown in the following equation:

$$y_{i,t}=\alpha +\mu_{i}+\lambda_{t}+\theta treat_{i}\times post_{i,t}+\beta x_{i,t}+\varepsilon_{i,t}$$ (1)

$$y_{i,t}=\alpha +\mu_{i}+\lambda_{t}+\theta Did+\beta x_{i,t}+\varepsilon_{i,t}$$ (2)

$$y_{i,t-1}=\alpha +\mu_{i}+\lambda_{t-1}+\theta treat_{i}\times post_{i,t-1}+\beta x_{i,t-1}+\varepsilon_{i,t-1}$$ (3)

$$y_{i,t-1}=\alpha +\mu_{i}+\lambda_{t-1}+\theta {Did}_{t-1}+\beta x_{i,t-1}+\varepsilon_{i,t-1}$$ (4)

In Eq. 1, y_i,t_ is the dependent variable referring to the self-reported health of subject i in period t; x_i,t_ represents a set of confounding factors including city level and individual level; treat_i_×post_i,t_ is the product term of the treatment dummy variable group and the time dummy variable; θ is the net effect of air control on health; µ_i_ represents the individual fixed effect; λ_t_ represents the time fixed effect; ɛ_i,t_ is the random error term. We used Did replace treat_i_×post_i,t_ in Eq. 2. In Eq. 3, the definition is the same as in Eq. 1. We used Did_t-1_ to replace treat_i_×post_i,t-1_ in Eq. 4. However, we used the PM_10_ and PM_2.5_ in the forward year to replace the current year, meaning that we could estimate the effect of air pollution control on health with a one-year lag.

The degree of income-related health inequality was calculated using the concentration index (CI). The CI was first introduced by Wagstaff et al., and has been widely applied as a standard method to describe and measure the degree of income-related inequality in various measures of health and healthcare utilization [3, 16, 29–33] The CI value ranges from −1 to 1. The positive value of the CI represents that good health is more concentrated in higher-income groups and *vice versa*. A formula for computing the concentration index is:

$$C=\frac{2}{\mu }\text{cov}\left(y_{i},R_{i}\right)$$ (5)

where C is the concentration index, $y_{i}$ refers to self-reported health, μ is the mean health of the entire population, and $R_{i}$ symbolizes the relative fractional rank of the economic status distribution.

The degree of income-related health inequity was calculated using the horizontal inequity index. Income-related inequality in health does not imply health inequity [34]. The horizontal inequity (HI) of health indicates the inequality in health by subtracting the contribution of need variables. The HI index is obtained by subtracting the contribution of unavoidable variables (e.g., sex and age) from the concentration index [35]. The HI index is positive, which signifies the existence of a pro-rich inequity (and *vice versa* in the case of a negative HI index).

## Results

The study population included 12,171 respondents (9,210 [47.36%] women; 10,236 [52.64%] men) in panel A (Air pollution controls measured by PM_10_), and 19,446 respondents (5,787 [47.55%] women; 6,384 [52.45%] men) in panel B (environmental controls identified by PM_2.5_). The majority of respondents were >64 years old, married, educated to the primary school level, employed, had basic health insurance, and reported a diagnosed chronic disease. Most residents had fair, poor, and very poor self-reported health (Supplementary Table S3).

**FIGURE 2:**
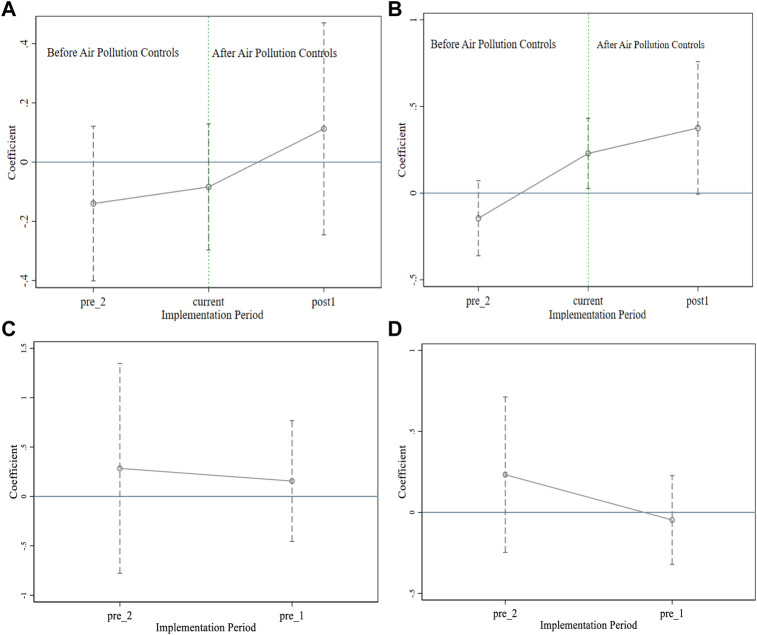
Common trend analysis with the effect of air pollution controls identified by PM_10_ and PM_2.5_ on health (Impact of Air Pollution Controls, Shaanxi, China, 2024). Note: **(A)** shows the common trend of the effect of air pollution controls identified by PM_10_ on health. **(B)** Shows the common trend of the effect of air pollution controls with a one-year lag identified by PM_10_ on health. **(C)** Shows the common trend of the effect of air pollution controls identified by PM_2.5_ on health. **(D)** Shows the common trend of the effect of air pollution controls with a one-year lag identified by PM_2.5_.

Figure 2 shows the common trends before the intervention of air pollution controls identified by PM_10_ and PM_2.5_. All the pre-intervention period estimates in panels A and B are not statistically significant. Plotting the self-reported health trajectories of the control and treatment groups for the pre-intervention periods revealed no substantial differences between the two groups (Supplementary Table S4). For the subjects with a one-year intervention lag, there were significant upward trends after the introduction of air pollution control. Overall, the results are consistent with the common trend hypothesis.

The results of the DID analysis are summarized in Table 1. For panel A and panel B, all the estimates for air pollution control without a one-year lag are not statistically significant, while the estimates for the subjects with a one-year lag are significantly positive (Table 1). Compared with the control group, respondents are 20% (OR 1.20, 95% *CI*, 1.02–1.42) and 24% (OR 1.24, 95% *CI*, 1.03–1.58) more likely to have very good and good health after air pollution controls in panels A and B, respectively. Table 1 shows that air pollution controls had a positive effect on self-reported health. The DID results indicate that the odds of respondents reporting very good and good health are 26% (95% *CI*, 1.03–1.54) and 38% (95% *CI*, 1.10–1.83) in the current year of the intervention with a one-year lag in panels A and B, respectively. The DID results indicate that the odds of respondents reporting very good and good health are 46% (95% *CI*, 1.04–2.13) and 83% (95% *CI*, 1.13–2.97) after 3 years of intervention with a one-year lag in panels A and B, respectively. Overall, the positive effect of air pollution controls on health presents an upward trend.

**TABLE 1:** The effect of environmental controls on health and time trend analysis (Impact of Air Pollution Controls, Shaanxi, China, 2024).

	Model 1	Model 2
Panel A		
Did	0.92 (0.77–1.10)	0.86 (0.71–1.03)
Current year of air pollution control	0.99 (0.81–1.21)	0.92 (0.74–1.14)
3 years after air pollution control	1.23 (0.87–1.73)	1.12 (0.78–1.60)
Did_t-1_	1.21 (1.02–1.42)**	1.20 (1.02–1.42)**
Current year of air pollution control	1.30 (1.07–1.58)***	1.26 (1.03–1.54)**
3 years after air pollution control	1.51 (1.05–2.17)**	1.46 (1.04–2.13)**
Control variables	No	Yes
Time-fixed effect	Yes	Yes
Individual fixed effect	Yes	Yes
Observation	19,446	19,446
Panel B		
Did	0.96 (0.58–1.59)	0.92 (0.54–1.53)
Current year of air pollution control	0.96 (0.72–1.29)	0.93 (0.69–1.26)
3 years after air pollution control	1.08 (0.65–1.78)	1.13 (0.67–1.90)
Did_t-1_	1.22 (0.99–1.53)*	1.24 (1.03–1.58)**
Current year of air pollution control	1.43 (1.11–1.86)***	1.38 (1.10–1.83)***
3 years after air pollution control	1.99 (1.27–3.10)***	1.83 (1.13–2.97)***
Control variables	No	Yes
Time-fixed effect	Yes	Yes
Individual fixed effect	Yes	Yes
Observation	12,171	12,171

Note: Model 1 means the results without control variables, and Model 2 means the results with control variables. Panel A means air pollution controls identified by PM_10_, and Panel B means air pollution controls identified by PM_2.5_. Did means the interaction term (Treat*post) between the air pollution controls and year dummy variables. Did_t-1_, means the interaction term (Treat*post _t-1_) between the air pollution controls and one-year lag dummy variables. Did is the net effect of air pollution control on health, and Did_t-1_, means the net effect of air pollution control with a one-year lag on health. *p< 0.1; **p< 0.05; ***p< 0.01.

Table 2 shows the effects and time-trend effects of economic status. We noted no significant effects on the health of the poorer and middle groups in the intervention group compared to the control group. Air pollution controls significantly improved the health of the poorest group. The DID trend effects analysis indicates that the poorest respondents are 41% more likely to report very good and good health in the current intervention period (OR 1.41, 95% *CI*, 1.01 to 2.08 in panel A). Air pollution controls improve the health of the richest group in panel B, but have insignificant effects in panel A. Thus, air pollution controls have a positive and lasting upward effect on the poorest group.

**TABLE 2:** The effect of air pollution controls on health for subjects with different economic statuses (Impact of Air Pollution Controls, Shaanxi, China, 2024).

	Economic status				
	Poorest group	Poorer group	Medium group	Richer group	Richest group
Panel A					
Did	1.23 (0.51–2.95)	0.32 (0.15–0.70)***	0.89 (0.40–1.98)	0.31 (0.16–0.63)***	0.76 (0.44–1.32)
Current year of air pollution control	1.08 (0.31–3.75)	0.34 (0.14–0.81)**	1.06 (0.42–2.64)	0.22 (0.08–0.54)***	0.76 (0.40–1.43)
3 years after air pollution control	3.11 (0.31–30.91)	0.45 (0.10–2.03)	1.01 (0.20–5.15)	0.10 (0.02–0.49)***	0.73 (0.25–2.13)
Did_t-1_	1.41 (1.01–2.08)**	0.88 (0.42–1.84)	0.52 (0.23–1.12)*	0.98 (0.53–1.84)	1.36 (0.85–2.20)
Current year of air pollution control	1.44 (0.98–2.12)*	0.83 (0.38–1.80)	0.53 (0.24–1.18)	0.89 (0.45–1.76)	1.43 (0.82–2.53)
3 years after air pollution control	2.45 (1.02–7.07)**	0.66 (0.16–2.65)	0.62 (0.16–2.42)	0.68 (0.20–2.25)	1.58 (0.58–4.31)
Controls	Yes	Yes	Yes	Yes	Yes
Time effect	Yes	Yes	Yes	Yes	Yes
Individual effect	Yes	Yes	Yes	Yes	Yes
Observation	3,885	3,886	3,886	3,886	3,885
Panel B					
Did	2.02 (0.43–9.56)	0.65 (0.16–2.50)	0.14 (0.04–0.45)**	0.85 (0.32–2.27)	1.16 (0.53–2.53)
Current year of air pollution control	1.50 (0.65–3.45)	0.57 (0.27–1.21)	0.47 (0.26–0.85)**	0.95 (0.58–1.56)	1.10 (0.74–1.63)
3 years after air pollution control	0.45 (0.08–2.35)	4.18 (1.02–17.14)**	2.09 (0.14–11.58)	1.01 (0.38–2.66)	0.81 (0.37–1.75)
Did_t-1_	4.48 (1.32–15.18)**	0.50 (0.16–1.53)	0.78 (0.30–2.07)	1.86 (0.82–4.22)	2.12 (1.09–4.13)**
Current year of air pollution control	5.07 (1.45–17.73)***	0.60 (0.17–2.11)	0.75 (0.26–2.23)	2.15 (0.26–2.23)	3.76 (1.09–4.13)***
3 years after air pollution control	16.65 (1.01–278.22)**	0.93 (0.11–7.82)	0.68 (0.11–4.28)	2.91 (0.26–2.23)	9.83 (2.0–48.28)***
Controls	Yes	Yes	Yes	Yes	Yes
Time effect	Yes	Yes	Yes	Yes	Yes
Individual effect	Yes	Yes	Yes	Yes	Yes
Observation	2,432	2,432	2,433	2,432	2,432

Note: Panel A means air pollution controls identified by PM_10_, and Panel B means air pollution controls identified by PM_2.5_. Did means the interaction term (Treat*post) between the air pollution controls and year dummy variables. Did_t-1_, means the interaction term (Treat*post _t-1_) between the air pollution controls and one-year lag dummy variables. Did is the net effect of air pollution control on health, and did_t-1_, indicates the net effect of air pollution control with a one-year lag on health. *p< 0.1; **p< 0.05; ***p< 0.01.

We performed a counterfactual analysis to test the robustness of the results. We assumed that the air pollution controls were implemented ahead of schedule. Supplementary Table S5 shows that none of the estimates from the counterfactual analysis are significant for all participants and different economic status groups, illustrating that the results we obtained earlier are robust. We only used 2013 and 2015 data to estimate the effect (see Supplementary Table S6), and the results indicate that our findings are robust. We selected some subjects in the control group as the treatment group (see Supplementary Table S7), and the estimate is insignificant, illustrating that the results are robust. We also changed the control group and randomly dropped three cities from it. The estimate is similar to the main results, which demonstrates that the results are reliable.

The CIs and HIs of different groups in different years are presented in Table 3. All the CIs were positive, meaning that there is a statistically pro-rich health inequity and that excellent health is more concentrated in the rich economic class. The CIs increased with the years in the control group, while those for the intervention groups decreased after the air pollution controls. After subtracting the contributions of health need variables from the CIs, all HIs were positive, which means that there is a pro-rich health inequity. After the air pollution controls, the HI index in the intervention group decreased from 0.101 to 0.090 in 2013 and 2018, respectively (panel A). The HI index in the intervention group treated in 2015 decreased from 0.090 to 0.085 in 2013 and 2018, respectively (panel B). The HI index increased from 0.020 to 0.096 in the control group. The increase in pro-rich inequity in the intervention group treated in 2018 (0.089, 0.098, and 0.099) was reduced after air pollution controls (panel A). The HI index decreased after air pollution controls in the intervention group treated in 2018.

**TABLE 3:** Horizontal inequity of self-reported health for the different groups at different times with a one-year lag (Impact of Air Pollution Controls, Shaanxi, China, 2024).

	Control group			Intervention group treated in 2015			Intervention group treated in 2018		
	2013	2015	2018	2013	2015	2018	2013	2015	2018
Panel A
Contribution of need variables (age-sex)	0.050	0.019	0.027	0.024	0.011	0.030	0.020	0.033	0.032
Contribution of control variables	0.197	0.425	0.479	0.364	0.344	0.370	0.354	0.418	0.428
Residual	−0.177	−0.320	0.384	−0.263	−0.244	−0.280	−0.265	−0.320	−0.329
CI	0.071***	0.125***	0.124***	0.124***	0.111***	0.120***	0.109***	0.130***	0.131***
HI	0.020	0.106	0.096	0.101	0.099	0.090	0.089	0.098	0.099
Observation	713	713	713	1,983	1,983	1,983	3,786	3,786	3,786
Panel B
Contribution of need variables (age-gender)				0.030	0.026	0.025	−0.016	0.028	0.044
Contribution of control variables				0.348	0.363	0.335	0.327	0.335	0.364
Residual				−0.258	−0.268	−0.249	−0.215	−0.254	−0.294
CI				0.120***	0.121***	0.110***	0.096***	0.109***	0.113***
HI				0.090	0.095	0.085	0.112	0.088	0.069
Observation				2,473	2,473	2,473	1,584	1,584	1,584

Note: Control group means that the subjects of the city’s annual average concentration of PM_10_ did not meet the interval released by the WHO. Intervention group in 2015 means that subjects living in the cities that implemented the air pollution controls in 2015 (annual average concentration of PM_10_ or PM_2.5_ decreased by 20 ug/m^3^ and 10 ug/m^3^ in 2015). Intervention group in 2018 means that subjects living in the cities that implemented the air pollution controls in 2018 (annual average concentration of PM_10_ or PM_2.5_ decreased by 20 ug/m^3^ and 10 ug/m^3^ in 2018). Because all the cities’ annual average concentration of PM_2.5_ decreased by 10 ug/m^3^ during 2013–2018, there was no control group and the values of the control group in panel B were missing. ***p< 0.01.

## Discussion

Our study examined the impact of air pollution controls on health and health inequity in China using the concentration index and the horizontal index for the first time. Our findings have shown that the control of air pollution is effective for health, and there is a lagged and lasting positive effect on health. Another interesting point is that air pollution controls generate the largest effect on the poorest population. We also noted that there are pro-rich health inequities in air pollution controls. Health inequity was reduced after air pollution controls, which means that the AQG set by the WHO can encourage China to reduce health risks and inequity from air pollution.

Although substantial studies focus on the relationship between air pollution and health outcomes, very few studies focus on air quality controls and the long-term impact of air pollution controls on health through longitudinal surveys, especially in older people. Our research offers new inspiration for other developing countries to manage air quality and achieve health equity. The current study found that air pollution controls contribute to improved health and that the positive effect is delayed by 1 year, which is consistent with earlier studies conducted in other countries like the United Kingdom. Previous studies have found that people exposed to green spaces may have health benefits by engaging in beneficial physical activity and ameliorating their stress response. This study is also consistent with the previous research in Europe [36], which indicated that the implementation of emission abatement strategies produces positive effects on the reduction of multiple-pollutant concentrations and that air quality improvement policies have beneficial effects on health. We also found that the impact of air pollution controls increases with the time of implementation. The implications of the study are clear: environmental controls could be crucial in the fight to improve health over the long term. AQG has a positive impact on air pollution controls and improves health.

The greatest health benefits from improvements in PM_10_ and PM_2.5_ levels are obtained by people with the lowest socioeconomic status. This is in line with published work exploring the relationship between socioeconomic status, air pollution, and health. The possible reason is that poor individuals are more vulnerable to air pollution and have the highest exposure due to work environments such as outdoor work and more contaminated occupations [3], and they are more likely to be exposed to pollutants from indoor heating and cooking. Another possible mechanism is that they have limited options for self-protection against air pollution, such as wearing masks and buying air purifiers. So, the poorest people are more sensitive to environmental changes and air pollution controls than the rich group. On the other hand, the health of the poorest groups is relatively bad, making them more vulnerable to health damage from air pollution than the general population.

The notion in our study that interventions at the societal level, such as improving the air quality where people live, could impact health inequalities is novel. We estimated trends in health inequities before and after air pollution controls and noted that the pro-rich health inequity decreased with the improvement in air pollution. Conversely, populations living in areas without improvements in air quality might observe an increase in income-related health inequity, which could have implications for those developing countries where environmental change remains a major challenge to achieving the SDGs. In the United Kingdom, Richard et al. conducted a cross-sectional survey and found that people living in green areas had lower inequality in mortality from all causes and circulatory diseases than those living in areas with less exposure to green spaces [37]. Greater exposure to air pollution is a driver of health inequalities found among people of low socioeconomic status [21]. The reason is that if we reduce the same amount of exposure to environmental hazards through air quality controls, the differences in vulnerability and avoidance of air pollution among populations of different socioeconomic statuses will be reduced.

This study has several limitations. First, due to the limited air quality data in China, we only used city-level data to perform the analysis. Second, the dependent variable used in this study is subjective, and we need to extend the study to objective health indicators. Third, we did not include indoor air pollution controls. Fourth, our conclusion may not be generalizable to the general population, as our sample is limited. Finally, the present study was subject to possible unobserved confounding factors, such as disability status, indoor air pollution, and so on.

Air pollution controls improved Chinese self-reported health and health equity, and the positive impacts increased year by year, which is in line with the achievement of SDG goals and AQG. The largest effects of air pollution controls were observed among the poorest population. After the air pollution controls, the concentration index and the horizontal inequity index decreased. Therefore, it is imperative for air-polluted regions to urgently foster AQG, increase government investment in air pollution controls, and scale up environmental actions to reduce the population’s exposure to air pollution by reducing the health damage and health inequity caused by air pollution. In addition, promoting equal access to basic public services, focusing on environmental protection, and improving the ability of vulnerable groups to prevent health risks are also key policies to improve health equity [35].

## Data Availability

Data can be obtained from the China Health and Retirement Longitudinal Study website.

## Abbreviations

SDGs: Sustainable Development Goals; WHO: World Health Organization; AQG: Air Quality Guidelines; PM_2.5_: fine particulate matter ≤2.5; PM_10_: inhalable particulate matter with a diameter of 10 μm; GDP: gross domestic product; CHARLS: China Health and Retirement Longitudinal Study; DID: Difference-in-differences; CI: Concentration index; HI: horizontal inequity.
