# Supplementary material for: The Impact of Air Pollution Controls on Health and Health Inequity Among Middle-Aged and Older Chinese: Evidence From Panel Data
Source: Int J Public Health. 2024 Jun 14;69:1606956. doi: 10.3389/ijph.2024.1606956 (PMC11211253; doi:10.3389/ijph.2024.1606956)
Supplement: Supplementary file 1 [file DataSheet1.docx]

# Supplemental Materials

**Table S1** The targets for PM_2.5_ and PM_10_ from the WHO, 2005 (Impact of Air Pollution Controls, Shaanxi, China. 2024)

| Interim targets | Annual PM_10_ | Annual PM_2.5_ |
| --- | --- | --- |
| First stage | 70ug/m3 | 35ug/m3 |
| Second stage | 50ug/m3 | 25ug/m3 |
| Third stage | 30ug/m3 | 15ug/m3 |

**Table S2** The targets for PM_2.5_ and PM_10_ from the WHO,2021 (Impact of Air Pollution Controls, Shaanxi, China. 2024)

| Interim targets | Annual PM_10_ | Annual PM_2.5_ |
| --- | --- | --- |
| First stage | 70ug/m3 | 35ug/m3 |
| Second stage | 50ug/m3 | 25ug/m3 |
| Third stage | 30ug/m3 | 15ug/m3 |
| Fourth stage | 20ug/m3 | 10 ug/m3 |

**Table S3** Variable definitions and summary statistics (Impact of Air Pollution Controls, Shaanxi, China. 2024)

| Variables | Description | Panel A-All residents  (*N*=19,446) | Panel B-All residents  (*N*=12,171) |
| --- | --- | --- | --- |
| **Control variables** |  |  |  |
| **Individual level** |  |  |  |
| Sex | 0=Female subjects | 9,210 (47.36) | 5,787 (47.55) |
|  | 1=Male subjects | 10,236 (52.64) | 6,384 (52.45) |
| Age (years) | 1=45-54(years) | 4,264 (21.93) | 2,625 (21.57) |
|  | 2=55-64(years) | 7,375 (37.93) | 4,689 (38.53) |
|  | 3=>64(years) | 7,807 (40.15) | 4,857 (39.91) |
| Education status^a^ | 1=Illiterate | 4,413 (22.69) | 2,440 (20.05) |
|  | 2=Primary school | 8,266 (42.51) | 5,390 (44.29) |
|  | 3=Junior middle school | 4,383 (22.54) | 2,743 (22.54) |
|  | 4=Senior high school | 2,006 (10.32) | 1,336 (10.98) |
|  | 5=College degree or above | 377 (1.94) | 261 (2.14) |
| Marital status | 1=Unmarried | 132 (0.68) | 90 (0.74) |
|  | 2=Married | 17,022 (87.53) | 10,669 (87.66) |
|  | 3=Divorced or widowed | 2,292 (11.79) | 1,412 (11.60) |
| Economic level^a^ | 1=Poorest | 259.68 (183.56) | 253.21 (185.02) |
|  | 2=Poor | 1676.64 (731.98) | 1694.24 (735.79) |
|  | 3=Medium | 5480.84 (1489.49) | 5513.99 (1492.29) |
|  | 4=Richer | 13560.93 (3483.41) | 13694.48 (3452.07) |
|  | 5=Richest | 60886.34 (53054.18) | 63160.74 (54658.16) |
| Work status^a^ | 1=Employed | 12,322 (63.43) | 7,464 (61.38) |
|  | 2=Unemployed | 304 (1.56) | 230 (1.89) |
|  | 3=Retired | 2,072 (10.67) | 1,416 (11.64) |
|  | 4=Jobless | 4,729 (24.34) | 3,050 (25.08) |
| Health insurance^a^ | 1=Uninsured | 779 (4.02) | 481 (3.97) |
|  | 2=Basic health insurance | 17,446 (90.14) | 10,859 (89.62) |
|  | 3=Commercial health insurance | 466 (2.41) | 304 (2.51) |
|  | 4=Others | 664 (3.43) | 473 (3.90) |
| Chronic disease^a^ | 0=No | 5,170 (26.68) | 3,443 (28.41) |
|  | 1=Yes | 14,207 (73.32) | 8,677 (71.59) |
| **City level** |  |  |  |
| Per capita GDP in each city | Continuous variables | 10.82 (0.53) | 11.01 (0.49) |
| The number of hospitals in each city ^a^ | Continuous variables | 262.47 (230.81) | 299.92 (260.53) |
| **Outcome variable** |  |  |  |
| Self-reported health | 1=Very good and good | 4,846 (24.92) | 3,295 (27.07) |
|  | 0=Fair, Poor, and very poor | 14,600 (75.08) | 8,876 (72.93) |

Note: Means (SD) were performed for continuous variables. *n* (%) were performed for categorical variables. ^a^ There were missing values. For panel A, there were 1 missing value in education status, 11 missing values in work status, 54 missing values in health insurance, and 51 missing values in chronic disease. For panel B, there were 1 missing value in education status, 19 missing values in work status, and 91 missing values in health insurance, 69 missing values in chronic disease.

**Table S4** Common trend tests (Impact of Air Pollution Controls, Shaanxi, China. 2024)

|  | Model 1 | Model 2 | |
| --- | --- | --- | --- |
| **Panel A** | | | |
| Did |  |  | |
| 2 years before air pollution controls | 0.84 (0.65 to 1.08) | 0.87 (0.67 to 1.13) | |
| Current year of air pollution controls | 0.99 (0.81 to 1.21) | 0.92 (0.74 to 1.14) | |
| 3 years after air pollution controls | 0.88 (0.88 to 1.73) | 1.12 (0.78 to 1.60) | |
| Did_t-1_ |  |  | |
| 2 years before air pollution controls | 0.86 (0.69 to 1.06) | 0.87 (0.67 to 1.13) | |
| Current year of air pollution controls | 1.30 (1.07 to 1.58)*** | 1.26 (1.03 to 1.54)** | |
| 3 years after air pollution controls | 1.51 (1.05 to 2.17)** | 1.46 (1.04 to 2.13)** | |
| Control variables | No | Yes | |
| Time-fixed effect | Yes | Yes | |
| Individual fixed effect | Yes | Yes | |
| Observation | 19,446 | 19,446 | |
| **Panel B** | | |  |
| Did |  |  | |
| 5 years before air pollution controls | 1.16 (0.42 to 3.19) | 1.33 (0.46 to 3.84) | |
| 2 years before air pollution controls | 1.08 (0.60 to 1.93) | 1.16 (0.63 to 2.15) | |
| Did_t-1_ |  |  | |
| 5 years before air pollution controls | 1.33 (0.85 to 2.08) | 1.26 (0.78 to 2.04) | |
| 2 years before air pollution controls | 0.96 (0.74 to 1.25) | 0.95 (0.73 to 1.25) | |
| Control variables | No | Yes | |
| Time-fixed effect | Yes | Yes | |
| Individual fixed effect | Yes | Yes | |
| Observation | 12,171 | 12,171 | |

Note: ***p*<0.05; ****p*<0.01.

**Table S5** Placebo tests (Based on the counterfactual assumption that the intervention was done one period earlier) (Impact of Air Pollution Controls, Shaanxi, China. 2024)

|  | All participants | Economic status | | | | |
| --- | --- | --- | --- | --- | --- | --- |
|  |  | Poorest group | Poorer group | Medium group | Richer group | Richest group |
| **Panel A** | | | | | | |
| Did_t-1_ | 1.14 (0.85 to 1.57) | 1.62 (0.29 to 2.98) | 0.98 (0.19 to 1.48) | 1.26 (0.78 to 2.57) | 3.14 (0.74 to 13.44) | 1.69 (0.33 to 2.84) |
| Controls | Yes | Yes | Yes | Yes | Yes | Yes |
| Time effect | Yes | Yes | Yes | Yes | Yes | Yes |
| Individual effect | Yes | Yes | Yes | Yes | Yes | Yes |
| Observation | 7,572 | 1,690 | 1,647 | 1,688 | 1,551 | 981 |
| **Panel B** | | | | | | |
| Did_t-1_ | 0.81 (0.58 to 1.13) | 0.64 (0.52 to 7.89) | 2.75 (0.44 to 16.93) | 0.64 (0.21 to 1.97) | 1.51 (0.46 to 4.90) | 0.38 (0.14 to 1.06) |
| Controls | Yes | Yes | Yes | Yes | Yes | Yes |
| Time effect | Yes | Yes | Yes | Yes | Yes | Yes |
| Individual effect | Yes | Yes | Yes | Yes | Yes | Yes |
| Observation | 3,168 | 819 | 714 | 688 | 592 | 352 |

**Table S6** Robustness checks (using 2013 and 2015 data) (Impact of Air Pollution Controls, Shaanxi, China. 2024)

|  | All participants | Economic status | | | | |
| --- | --- | --- | --- | --- | --- | --- |
|  |  | Poorest group | Poorer group | Medium group | Richer group | Richest group |
| **Panel A** | | | | | | |
| Did_t-1_ | 1.17 (1.04 to 1.40)** | 1.62 (1.06 to 2.45)** | 1.04 (0.37 to 2.92) | 0.55 (0.19 to 1.61) | 0.85 (0.35 to 2.03) | 2.21 (0.93 to 5.24)** |
| Controls | Yes | Yes | Yes | Yes | Yes | Yes |
| Time effect | Yes | Yes | Yes | Yes | Yes | Yes |
| Individual effect | Yes | Yes | Yes | Yes | Yes | Yes |
| Observation | 12,964 | 3,471 | 2,695 | 2,736 | 2,385 | 1,659 |
| **Panel B** | | | | | | |
| Did_t-1_ | 1.35 (1.02 to 1.80)** | 6.49 (1.27 to 7.89)** | 0.28 (0.04 to 1.98) | 0.58 (0.20 to 1.97) | 2.15 (0.62 to 7.40) | 5.38 (1.58 to 6.06)*** |
| Controls | Yes | Yes | Yes | Yes | Yes | Yes |
| Time effect | Yes | Yes | Yes | Yes | Yes | Yes |
| Individual effect | Yes | Yes | Yes | Yes | Yes | Yes |
| Observation | 8,104 | 2,136 | 1,709 | 1,761 | 1,454 | 1,044 |

Note: The results just used the data from 2013 and 2015 to estimate the effect of air pollution controls on health. ***p*<0.05; ****p*<0.01.

**Table S7** Robustness checks (changing the control group in panel A) (Impact of Air Pollution Controls, Shaanxi, China. 2024)

|  | Model 1 | Model 2 |
| --- | --- | --- |
| Did_t-1_ | 1.13 (0.44 to 2.88) | 1.18 (1.04 to 1.40)** |
| Control variables | No | Yes |
| Time fixed effect | Yes | Yes |
| Individual fixed effect | Yes | Yes |
| Observation | 2,139 | 18,759 |

Note: Model 1 means that we choose one control city as the treatment group and treated in 2015. Model 2 means that we random drop three control cities from control group.***p*<0.05.
